# Inhibitory Effect of Diabetes on Proliferation of Vascular Smooth Muscle After Balloon Injury in Rat Aorta

**DOI:** 10.1155/EDR.2000.101

**Published:** 2000

**Authors:** Gunilla Dahlfors, Yun Chen, Bertil Gustafsson, Hans J. Arnqvist

**Affiliations:** 1 Division of Cellbiology Linköping University Linköping S-581 85 Sweden; 2 Division of Pathology Linköping University Linköping S-581 85 Sweden; 3 Division of Internal Medicine Linköping University Linköping S-581 85 Sweden

## Abstract

The effect of streptozotocin-induced diabetes on cell proliferation in rat aortic intima-media, as well as on local gene expression of transforming growth factor-*β*1 (TGF-*β*1) was studied. TGF-*β*1 mRNA was measured by solution hybridization and TGF-*β*1 protein by ELISA. Proliferation was measured by bromodeoxyuridine incorporation into DNA two days after balloon injury. All BrdU-labelled cells observed were smooth muscle cells. After a diabetes duration of 2 and 4 weeks, labelled cells were significantly fewer compared with controls. Circulating levels of total TGF-*β*1 were lowered in rats with 2 weeks diabetes. Although the balloon injury procedure by itself stimulated the gene expression of TGF-*β*1, no significant difference in TGF-*β*1 mRNA content between diabetic and control rats after injury was found. In conclusion: vascular smooth muscle proliferation *in vivo* is inhibited by the diabetic state in this model of insulin deficient diabetes and this inhibition is not related to an impaired local expression of TGF-*β*1.


*Int. Jnl. Experimental Diab. Res.*, Vol. 1, pp. 101–109
Reprints available directly from the publisher
Photocopying permitted by license only

© 2000 OPA (Overseas Publishers Association) N.V.
Published by license under
the Harwood Academic Publishers imprint,
part of The Gordon and Breach Publishing Group.
Printed in Malaysia.

### Inhibitory Effect of Diabetes on Proliferation of Vascular Smooth Muscle After Balloon Injury in Rat Aorta

GUNILLA DAHLFORS[a,*], YUN CHEN[a], BERTIL GUSTAFSSON[b] and HANS J. ARNQVIST[a,c]

[a] *Division of Cellbiology,* [b] *Division of Pathology,* [c] *Division of Internal Medicine, Linköping University, S-581 85 Linköping, Sweden*

*(Received in final form 6 February 2000)*

**The effect of streptozotocin-induced diabetes on cell proliferation in rat aortic intima-media, as well as on local gene expression of transforming growth factor-β1 (TGF-β1) was studied. TGF-β1 mRNA was measured by solution hybridization and TGF-β1 protein by ELISA. Proliferation was measured by bromodeoxyuridine incorporation into DNA two days after balloon injury. All BrdU-labelled cells observed were smooth muscle cells. After a diabetes duration of 2 and 4 weeks, labelled cells were significantly fewer compared with controls. Circulating levels of total TGF-β1 were lowered in rats with 2 weeks diabetes. Although the balloon injury procedure by itself stimulated the gene expression of TGF-β1, no significant difference in TGF-β1 mRNA content between diabetic and control rats after injury was found. In conclusion: vascular smooth muscle proliferation *in vivo* is inhibited by the diabetic state in this model of insulin deficient diabetes and this inhibition is not related to an impaired local expression of TGF-β1.**

*Keywords:* Angioplasty, de-endothelialization, DNA synthesis, streptozotocin, transforming growth factor-β1

### INTRODUCTION

In the development of atherosclerosis and in restenosis after balloon angioplasty, proliferation of vascular smooth muscle cells plays a central role.[1,2] Smooth muscle cell growth is regulated by different growth factors that are locally produced in the vessel wall or circulate in the blood. When proliferation of vascular smooth muscle cells is provoked by balloon angioplasty, there is an increased expression of growth factors such as platelet-derived growth factor (PDGF),[3] basic fibroblast growth factor (bFGF),[4] insulin-like growth factor-I (IGF-I)[5] and transforming growth factor-β1 (TGF-β1)[6,7] in the vessel wall.

It is well known that diabetes is associated with an increased risk of atherosclerotic disease.[8] During the last years there has been a

*Corresponding author. Tel.: 46 13 222789, Fax: 46 13 224273, e-mail: gunda@mcb.liu.se growing interest for the role of TGF-$\beta$1 in diabetes associated nephropathy, macrovascular complications [9] and wound healing.[10–13] High glucose has been shown to induce the expression of TGF-$\beta$1 in some cell types [9] and circulating levels of TGF-$\beta$1 are reported to be elevated in diabetic rats [14] and in NIDDM patients.[15] However, in wounds of diabetic rats with retarded wound healing, levels of TGF-$\beta$, bFGF and IGF-I are decreased[11, 13] and treatment with TGF-$\beta$ has been shown to improve wound healing in diabetic animals.[10–12]

Bornfeldt *et al.*, studied the effect of experimental diabetes on the response of arteries to injury and showed that $^3$H-thymidine incorporation into aortic media two days after balloon injury was inhibited in rats with streptozotocin-induced diabetes.[5] Another study reported an inhibited intima : media area ratio in the carotid artery after balloon injury in the same diabetic model.[16] In insulin treated diabetic BB (Bio breeding) Wistar rats, intima was reported to be thicker, with more smooth muscle cell layers than the intima of non-diabetic Wistar rats following balloon injury to the aorta.[17] In patients with type II diabetes mellitus, a greater incidence of restenosis after arterial injury caused by coronary stenting has been reported[18] while wound healing in general is impaired.[12, 19]

The present study was undertaken to investigate the effect of diabetes on early cell proliferation in the aorta after injury. We also investigated if the effect of diabetes was associated with a change in circulating levels of TGF-$\beta$1 or changes in local TGF-$\beta$1 gene expression.

### MATERIALS AND METHODS

### Animals

Male Sprague-Dawley rats (ALAB, Stockholm, Sweden) weighing about 200 g were used in this study. The rats were kept under 12 h light: 12 h darkness cycle and had free access to food and water. All animal experiments were made in agreement with the principles of laboratory animal care and were approved by the local ethics committee for animal experiments. Diabetes was induced by i.v. injection of streptozotocin (65 mg/kg body weight) in a tail vein, while rats injected with 0.9% NaCl served as controls. Blood samples were taken from tail vein and blood glucose was measured using a hexokinase method (Gluco-quant®) or ONE TOUCH® test strips for quantitative measurement of glucose in blood.[20] The rats were considered diabetic if random blood glucose concentration exceeded 15 mmol/l.

### De-endothelialization

De-endothelialization was performed, using a balloon catheter, according to the method of Capron *et al.*[21] In short, the rats were kept under light ether anaestesia and a deflated embolectomy catheter (Fogarty, size 2F, Baxter Medical) was introduced into the aorta through the left common carotid artery down to the level of the renal artery. The balloon was inflated with 40–50 µl distilled water, and withdrawn to the level of the diaphragm. After the passage of the diaphragm had been felt, additional 20–30 µl distilled water was inflated. This procedure was repeated three times and the carotid artery was then double ligated. Control rats were sham operated: the same procedure was carried out except that no catheter was introduced into the carotid artery.

### Measurement of DNA Synthesis

DNA synthesis was measured two days after injury. The rats were injected intraperitoneally with 5-bromo-2′-deoxyuridine (BrdU) 0.1 mg/g body weight. Two hours after injection, the animals were killed and the aortic segment between the left subclavian artery and the celiac artey was removed and immersed in ice-cold saline. Within 30 minutes, cross sections of the central part of the aortic segment were cut out and quickly frozen at $-70°C$. With a setting at 4 μm, cryostat sections were cut out and brought to chrome-gelatinized glass slides. The slides were fixed in 70% ethanol for 1 h and were allowed to dry. DNA was denatured with 1.5 mol/l NaOH for 90 sec followed by neutralisation in 0.1 mol/l $Na_2B_4O_7$ (pH 8.5) for 10 sec and rinsing twice in a phosphate buffer (PBS). After incubation with an anti-BrdU monoclonal antibody and a peroxidase-conjugated secondary antibody developed with diaminobenzidine as a chromogen, cells in the S-phase were easily visualized. DNA synthesis was expressed as number of labelled cells in a whole cross section of aortic media layer counted under an ordinary light microscope (Fig. 1). One cross section from each aorta was counted.

### Measurement of mRNA by a Solution Hybridization Assay

The intima-media was separated from adventitia according to the method of Wolinsky and Daly.[22] The intima-media of the aortas were homogenized in a glass–glass homogenizer for 30 s. Nucleic acids were extracted essentially as described by Durnam and Palmiter.[23] Samples were digested with proteinase K and extracted with phenol and chloroform. Nucleic acids were precipitated with ethanol. Total nucleic acids were measured by spectrophotometry. DNA content was measured by fluorimetry according to the method of Labarca and Paigen.[24]

The mRNA level of TGF-$\beta$1 was determined by a solution hybridization assay[23] using a [$^{35}$S]-UTP labelled RNA probe complementary to a 1kb fragment spanning the major coding region of the TGF-$\beta$1 precursor.[25] The probe was prepared according to the method of Melton *et al.*[26] and was hybridized to total nucleic acid (TNA) samples at 70°C for 20 h. Hybridization was performed in 40 μl of 0.6 M NaCl, 20 mmol/l Tris/HCl (pH 7.5), 4 mmol/l EDTA, 0.1% SDS, 0.75 mmol/l dithiothreitol (DTT), 25% formamide and 20000 cpm [$^{35}$S]-UTP labelled probe per incubation. The samples were exposed to RNases and the hybrids precipitated with 100 μl TCA (6 mol/l), collected on glass microfibre filters and radioactivity measured in a liquid scintillation counter (1214 Rackbeta; LKB). The radioactivity

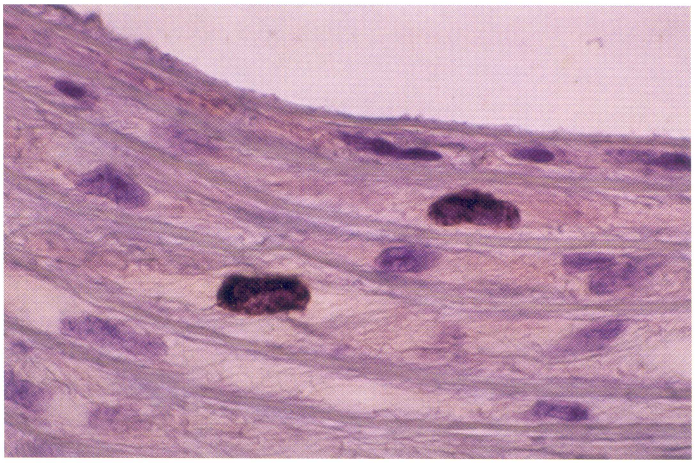

FIGURE 1 Photomicrograph of the media layer of aorta in a control rat, two days after balloon angioplasty to the aorta. No re-endothelialization of the injured aorta can be seen at this time point. The structure of the artery wall can be discerned and the nuclei of two smooth muscle cells in S-phase are strongly labelled by BrdU incorporation. (See Color Plate VI).

of each sample was then compared with a standard curve constructed from a sample with known amount of *in vitro* synthesized TGF-$\beta$1 sense RNA complementary to the probe. A standard curve was included in each assay and samples were analysed in triplicate. Tubes including only hybridization buffer and probe served as blanks.

### Immunoassay for TGF-$\beta$1

Serum levels of TGF-$\beta$1 were measured by a quantitative immunoassay kit for TGF-$\beta$1 (R&D Systems). Before analysis TGF-$\beta$1 protein was activated by acidification of serum samples. A standard curve made from recombinant TGF-$\beta$1 was included in each assay.

### Chemicals

Streptozotocin was obtained from Sigma Chemical Co. (St Louis, MO, USA) and ONE TOUCH® test strips were from Lifescan (Milpitas, California, USA). Gluco-quant®, 5-bromo-2'deoxyuridine, RNases, Proteinase K and herring sperm DNA were from Roche Diagnostics (Mannheim, Germany). Anti-BrdU monoclonal antibody and peroxidase-conjugated secondary antibody was purchased from Dakopatts AB (Hägersten, Sweden). Immunoassay kit for human TGF-$\beta$1 was obtained from R&D Systems (Abingdon, UK). [$^{35}$S]-UTP was from Amersham International (Amersham, Bucks, UK), chemicals for probe synthesis were received from Promega (Madison, WI, USA) and phenol was obtained from Fisher Scientific (Fair Lawn, NJ, USA).

### Statistics

Values are given as means $\pm$ SEM. Statistical comparisons were made according to analysis of variance (ANOVA) with Fisher's protected least significant difference (PLSD).

### RESULTS

Induction of diabetes was monitored by measuring body weight and blood glucose. Blood glucose was markedly elevated and body weight of streptozotocin injected rats was lower than in control rats (Tab. I).

Two days after balloon angioplasty the aortic intima-media was examined with regard to the presence of BrdU-labelled cells. At this time point no re-endothelialization of the injured aorta was seen and of the aortic intima-media only the media layer was present. All cells reacting with antibodies against BrdU were smooth muscle cells (Fig. 1). The number of labelled cells was lower in diabetic rats as compared with controls (Fig. 2). Proliferating cells in aortic media remained low in diabetic rats, while in nondiabetic rats, BrdU-labelled cells decreased along with the increased age. Consequently, the difference between control and diabetic rats was more pronounced in rats with a diabetes duration of 2 and 4 weeks than in rats with 8 weeks diabetes.

Total TGF-$\beta$1 was measured in serum from rats with a diabetes duration of 2 weeks (Fig. 3). Levels of TGF-$\beta$1 were slightly but significantly lower in diabetic animals as compared with controls. No difference was seen between balloon injured rats and sham operated rats in either control animals or diabetic animals. Levels of active TGF-$\beta$1 in serum was measured. A tendency to lower values of active serum TGF-$\beta$1 in diabetic rats as compared to controls was seen

TABLE I Body weight and blood glucose in normal and diabetic rats ($n = 3-4$)

| Diabetes duration (weeks) | Rat | Body weight (g) At STZ injection | At balloon injury | Blood glucose (mmol/l) |
|---|---|---|---|---|
| 2 | Control | $200 \pm 2$ | $313 \pm 8$ | $6.9 \pm 0.2$ |
| | Diabetes | $194 \pm 3$ | $187 \pm 8$ | $32.4 \pm 1.1$ |
| 4 | Control | $195 \pm 1$ | $360 \pm 10$ | $6.4 \pm 0.5$ |
| | Diabetes | $195 \pm 2$ | $206 \pm 5$ | $32.0 \pm 0.6$ |
| 8 | Control | $198 \pm 3$ | $476 \pm 9$ | $6.2 \pm 0.1$ |
| | Diabetes | $196 \pm 3$ | $240 \pm 18$ | $28.4 \pm 0.6$ |

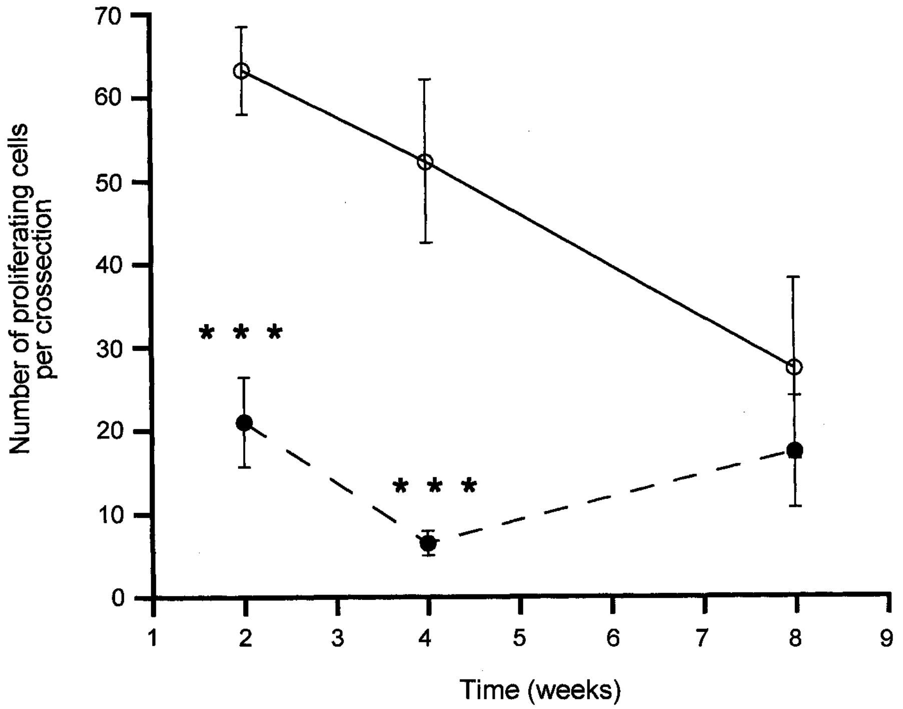

FIGURE 2 Effect of diabetes on proliferation of cells in rat aorta two days after balloon injury. Proliferation was measured by BrdU incorporation into DNA. Measurements were made at 2, 4 and 8 weeks of diabetes duration. Open circles represent non-diabetic control rats while filled circles represent diabetic rats. Bars are the mean $\pm$SEM ($n$=3–4). The ANOVA Fisher's PLSD was used for statistical comparisons: *** $p < 0.001$.

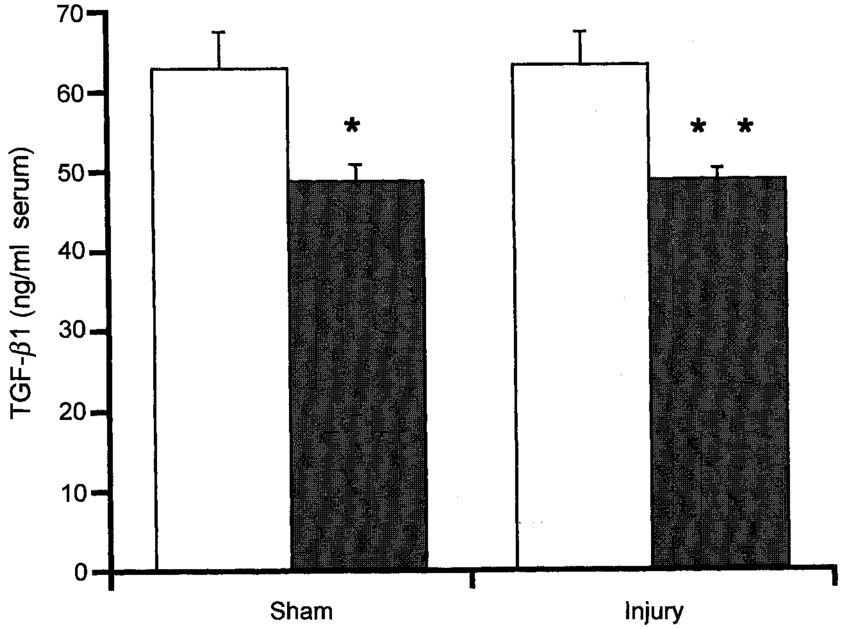

FIGURE 3 Serum levels of total TGF-$\beta$1 in control (open bars) and 2 week diabetic rats (solid bars) two days after balloon injury. Bars are the mean $\pm$SEM ($n = 5$). Statistical comparisons were made according to the ANOVA Fisher's PLSD: *$p < 0.05$, **$p < 0.01$.

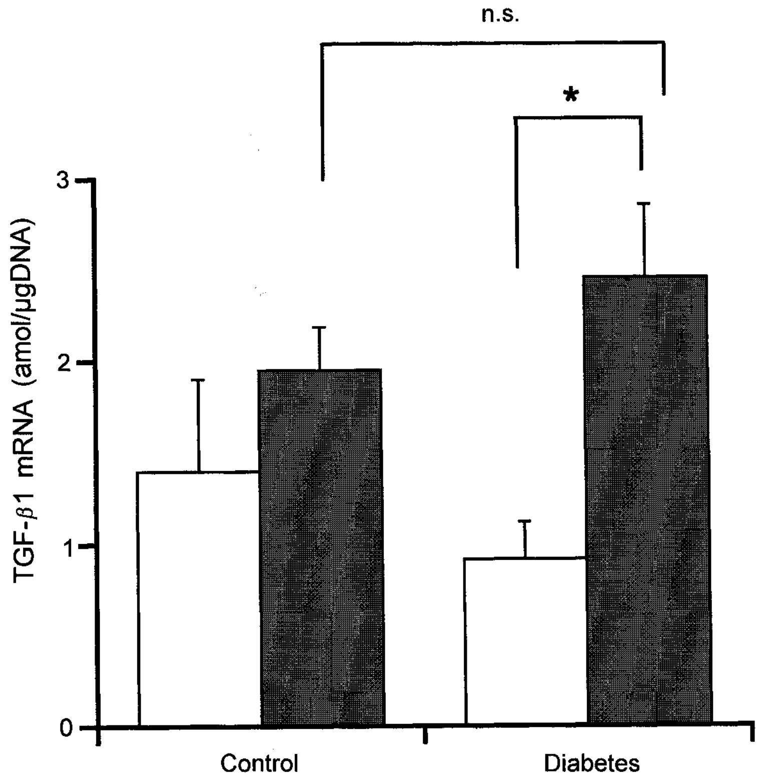

FIGURE 4 Expression of TGF-$\beta$1 mRNA in control and diabetic rat aorta two days after balloon injury (solid bars) or sham operation (open bars). Rats with a diabetes duration of 2 weeks were used for the experiment. Bars are the mean ± SEM ($n$=3–5). Statistical comparisons were made according to the ANOVA Fisher's PLSD: n.s.=not significant, * $p < 0.05$.

but values were below the lowest point in the standard curve and it was therefore not possible to get a quantitative measure of active TGF-$\beta$1.

TGF-$\beta$1 mRNA was measured after a diabetes duration of 2 weeks, in aorta of control and diabetic rats (Fig. 4). Basal level of TGF-$\beta$1 mRNA did not differ significantly between control and diabetic rats. In control rats TGF-$\beta$1 mRNA was 1.4 ± 0.5 amol/μg DNA and in diabetic rats, 0.92 ± 0.24 amol/μg DNA. There was an increased expression of TGF-$\beta$1 after balloon injury. TGF-$\beta$1 mRNA levels were increased from 1.4 ± 0.5 to 1.95 ± 0.24 amol/μg DNA in control rats and from 0.92 ± 0.35 to 2.46 ± 0.4 amol/μg DNA in diabetic rats after injury. No significant difference in TGF-$\beta$1 mRNA between injured diabetic aortas (2.46 ± 0.40 amol/μg DNA) and injured control (1.95 ± 0.24 amol/μg DNA) aortas was seen.

### DISCUSSION

It is known that proliferation of aortic cells induced by de-endothelialization peaks two days after injury.[21] We therefore determined BrdU-labelling two days after balloon catheter injury, a time point when the aorta was devoid of intima and there was no re-endothelialization. All BrdU-labelled cells were smooth muscle cells and the number of BrdU-labelled cells were lowered in diabetic aorta in comparison to aorta from nondiabetic control rats. Bornfeldt and coworkers[5] showed that $^3$H-thymidine incorporation into DNA was inhibited in aorta of diabetic rats after balloon injury and that the DNA content was lower in diabetic aorta 2 weeks after injury. In this study and in the study of Bornfeldt *et al.*, untreated streptozotocin diabetic Sprague-Dawley rats, an insulin deficient model of diabetes, was used. Another study using the same rat model reported an inhibited neointima formation in the carotid artery after balloon injury.[16] Our results show that inhibition of smooth muscle cell proliferation is a feature of the inhibited arterial response to injury in aorta of streptozotocin diabetic rats. In line with these results it was recently reported that the hypercellularity of the intima is reduced in restenotic tissue from diabetic patients while collagen-rich sclerotic content is increased.[27] It was suggested that an accelerated fibrotic rather than a proliferative response is the main feature of restenosis in diabetic patients.

In order to see whether diabetes duration had an effect on proliferation of smooth muscle cells, BrdU incorporation was measured at 2, 4 and 8 weeks after induction of diabetes. The proliferative response of smooth muscle cells in the intima-media remained low in rats with different diabetes duration without any significant change with time. Because, in our study, the proliferation of smooth muscle cells in normal rats decreased along with increased age, the difference between controls and diabetic rats was most pronounced 2 and 4 weeks after induction of diabetes. It has been reported that proliferation of vascular smooth muscle cells is enhanced by increased age.[28] This does not explain the observed decline in smooth muscle cell proliferation in normal rats in this study. The proliferative response of smooth muscle cells has been shown to be dependent on the severity of the arterial injury.[7] It is possible that the control rats at 8 weeks diabetes duration, which were considerably bigger than the control rats at 4 weeks of diabetes duration, were less injured by the balloon angioplasty procedure and thus responded with lower proliferation than expected.

It might be argued that streptozotocin by itself causes the impaired proliferation of smooth muscle cells in diabetic rat aorta. However, earlier studies in our laboratory showed that effects of streptozotocin induced diabetes on $H^3$-thymidine incorporation[5] and $C^{14}$-leucine incorporation[29] in rat aorta, could be reversed by insulin treatment. This suggests that the impaired metabolism and growth seen in streptozotocin diabetic rat aortas, is caused by the diabetic state and not by a toxic effect of streptozotocin.

TGF-$\beta$1 is an important regulator of vascular smooth muscle cell growth and is reported to stimulate smooth muscle cell proliferation after arterial balloon injury.[6,7] It has, however, also been stated that TGF-$\beta$1 might inhibit smooth muscle cell proliferation *in vivo*.[30] Expression of TGF-$\beta$1 is correlated with increased extracellular matrix production and neointima formation.[6,31,32] In wounds, low levels of TGF-$\beta$ are considered to contribute to the impaired healing seen in diabetes.[10–13] The role of TGF-$\beta$1 in diabetic vascular diseases is, however, still mainly unknown. We investigated the possibility that the low proliferation of smooth muscle cells in diabetic rats two days after injury might be associated with altered levels of circulating TGF-$\beta$1 and/or a changed expression of TGF-$\beta$1 as compared with injured controls locally in the vessel wall. Levels of total circulating TGF-$\beta$1 was slightly but significantly lower in balloon injured diabetic rats as compared to balloon injured control rats. The same pattern was found in sham operated rats. Active TGF-$\beta$1 in serum was measured but levels were below the lowest concentration in the standard curve. However, a tendency to lower levels of active TGF-$\beta$1 was seen in diabetic rats as compared to controls in agreement with results on total TGF-$\beta$1. In contrast to these results Bollinenni and colleagues found elevated levels of circulating active TGF-$\beta$1 in diabetic rats 6

weeks after induction by streptozotocin and levels were markedly higher than we found in this study.[14] The reason for such a discrepancy is not evident but might be partly due to the different methods used for measurement of TGF-$\beta$1, enzyme-linked immunosorbent assay in our study and a mink-lung epithelial cell bioassay in the study of Bollinenni and colleagues. Also the diabetes duration was different, 2 weeks and 6 weeks respectively.

A previous study,[6] reported that TGF-$\beta$1 mRNA was elevated after balloon injury to the aorta of non-diabetic rats. We found a significant increase in TGF-$\beta$1 mRNA by balloon injury in diabetic rats. This increased gene expression of TGF-$\beta$1 might contribute to the proliferation of smooth muscle cells triggered by the balloon injury procedure.[6,7] TGF-$\beta$1 mRNA in diabetic rats after injury was not, however, decreased as compared to injured controls. Thus, the low proliferation of smooth muscle cells in diabetic rats after injury was not related to a significant impairment of TGF-$\beta$1 gene expression locally. However, other growth factors, as previously described for IGF-I,[5] might be of importance for regulating smooth muscle cell proliferation in diabetic rats after balloon injury.

In conclusion, balloon injury to rat aorta induced proliferation of vascular smooth muscle cells. The cell proliferation was impaired in streptozotocin diabetic rats. Although the injury procedure by itself increased TGF-$\beta$1 mRNA levels, no significant difference in TGF-$\beta$1 mRNA levels between injured diabetic rats and injured controls could be seen, suggesting that the inhibited smooth muscle cell proliferation in diabetic rats was not caused by an impaired local TGF-$\beta$1 mRNA expression.

### *Acknowledgments*

We are grateful to Mrs. R. Gidlöf and K. Hellander for their excellent technical assistance. Financial supports were obtained from the Swedish Medical Research Council (19x-4952), Swedish Diabetes Association, Barndiabetes fonden and JDF Wallenberg foundation (K98-99JD-12813-01A).

### *References*

[1] Ferns, G. A. A., Stewart-Lee, A. L. and Änggård, E. E. (1992). Arterial response to mechanical injury: balloon catheter de-endothelialization, *Atherosclerosis*, **92**, 89–104.

[2] Ross, R. (1993). The pathogenesis of atherosclerosis: a perspective for the 1990s, *Nature*, **362**, 801–809.

[3] Majesky, M. W., Reidy, M. A., Bowen Pope, D. F., Hart, C. E., Wilcox, J. N. and Schwartz, S. M. (1990). PDGF ligand and receptor gene expression during repair of arterial injury, *J. Cell Biol.*, **111**, 2149–2158.

[4] Lindner, V., Lappi, D. A., Baird, A., Majack, R. A. and Reidy, M. A. (1991). Role of basic fibroblast growth factor in vascular lesion formation, *Circ. Res.*, **68**, 106–113.

[5] Bornfeldt, K. E., Arnqvist, H. J. and Capron, L. (1992). *In vivo* proliferation of rat vascular smooth muscle in relation to diabetes mellitus insulin-like growth factor I and insulin, *Diabetologia*, **35**, 104–108.

[6] Majesky, M. W., Lindner, V., Twardzik, D. R., Schwartz, S. M. and Reidy, M. A. (1991). Production of transforming growth factor $\beta$1 during repair of arterial injury, *J. Clin. Invest.*, **88**, 904–910.

[7] Reidy, M. A. (1992). Factors controlling smooth-muscle cell proliferation, *Arch. Pathol. Lab. Med.*, **116**, 1276–1280.

[8] Semenkovich, C. F. and Heinecke, J. W. (1997). The mystery of diabetes and atherosclerosis: time for a new plot, *Diabetes*, **46**, 327–334.

[9] Yokoyama, H. and Deckert, T. (1996). Central role of TGF-$\beta$ in the pathogenesis of diabetic nephropathy and macrovascular complications: a hypothesis, *Diabetic Med.*, **13**, 313–320.

[10] Benn, S. I., Whitsitt, J. S., Broadley, K. N., Nanney, L. B., Perkins, D., He, L., Patel, M., Morgan, J. R., Swain, W. F. and Davidson, J. M. (1996). Particle-mediated gene transfer with transforming growth factor-beta1 cDNAs enhances wound repair in rat skin, *J. Clin. Invest.*, **98**, 2894–2902.

[11] Bitar, M. S. and Labbad, Z. N. (1996). Transforming growth factor-beta and insulin-like growth factor-I in relation to diabetes-induced impairment of wound healing, *J. Surg. Res.*, **61**, 113–119.

[12] Broadley, K. N., Aquino, A. M., Hicks, B., Ditesheim, J. A., McGee, G. S., Demetriou, A. A., Woodward, S. C. and Davidson, J. M. (1989–90). The diabetic rat as an impaired wound healing model: stimulatory effects of transforming growth factor-beta and basic fibroblast growth factor, *Biotechnol. Ther.*, **1**, 55–68.

[13] Moulin, V., Lawny, F., Barritault, D. and Caruelle, J. P. (1998). Platelet releasate treatment improves skin healing in diabetic rats through endogenous growth factor secretion, *Cell. Mol. Biol.*, **44**, 961–971.

[14] Bollineni, J. S. and Reddi, A. S. (1993) Transforming growth factor-$\beta$1 enhances glomerular collagen synthesis in diabetic rats, *Diabetes*, **42**, 1673–1677.

[15] Pfeiffer, A., Drewes, C., Middelberg-Bisping, K. and Schatz, H. (1996). Elevated plasma levels of transforming growth factor-$\beta$1 in NIDDM, *Diab. Care.*, **19**, 1113–1117.
[16] Edelman, E. R., Pukac, L. A. and Karnovsky, M. J. (1993). Protamine and protamine-insulins excerbate the vascular response to injury, *J. Clin. Invest.*, **91**, 2308–2313.
[17] Winocour, P. D., Richardson, M. and Kinlough-Rathbone, R. L. (1993). Continued platelet interaction with de-endothelialized aortae associated with slower re-endothelialization and more extensive intimal hyperplasia in spontaneously diabetic BB rats, *Int. J. Exp. Path.*, **74**, 603–613.
[18] Carrozza, J. P. Jr., Kuntz, R. E., Fishman, R. F. and Baim, D. S. (1993). Restenosis after arterial injury caused by coronary stenting in patients with diabetes mellitus, *Annal. Internal Med.*, **118**, 344–349.
[19] Stadelmann, W. K., Digenis, A. G. and Tobin, G. R. (1998). Impediments to wound healing, *Am. J. Surg.*, **176**, 39S–47S.
[20] Marks, V. and Dawson, A. (1965). Rapid stick method for determining blood glucose concentration, *Brit. Med. J.*, **1**, 293.
[21] Capron, L., Jarnet, J., Kazandjian, S. and Housset, E. (1986). Growth promoting effects of diabetes and insulin on arteries. An *in vivo* study of rat aorta, *Diabetes*, **35**, 973–978.
[22] Wolinsky, H. and Daly, M. M. (1970). A method for the isolation of intima-media samples from arteries, *Proc. Soc. Exp. Biol. Med.*, **135**, 364–368.
[23] Durnam, D. M. and Palmiter, R. D. (1983). A practical approach for quantitating specific mRNAs by solution hybridization, *Anal. Biochem.*, **131**, 385–393.
[24] Labarca, C. and Paigen, K. (1980). A simple, rapid and sensitive DNA assay procedure, *Anal. Biochem.*, **102**, 344–352.
[25] Qian, S. W., Kondaiah, P., Roberts, A. B. and Sporn, M. B. (1990). cDNA cloning by PCR of rat transforming growth factor $\beta$-1, *Nucleic Acids Res.*, **18**, 3059.
[26] Melton, D. A., Krieg, P. A., Rebagliati, M. R., Maniatis, T., Zinn, K. and Green, M. R. (1984). Efficient *in vitro* synthesis of biologically active RNA and RNA hybridization probes from plasmids containing bacteriophage SP6 promotor, *Nucleic Acids Res.*, **12**, 7035–7056.
[27] Moreno, P. R., Fallon, J. T., Murcia, A. M., Leon, M. N., Simosa, H., Fuster, V. and Palacios, I. F. (1999). Tissue characteristics of restenosis after percutaneous transluminal coronary angioplasty in diabetic patients, *J. Am. Coll. Cardiol.*, **34**, 1045–1049.
[28] Stemerman, M. B., Weinstein, R., Rowe, J. W., Maciag, T., Fuhro, R. and Gardner, R. (1982). Vascular smooth muscle cell growth kinetics *in vivo* in aged rats, *Proc. Natl. Acad. Sci.*, USA, **79**, 3863–3866.
[29] Arnqvist, H. J. and Dahlkvist, H. H. (1979). Effect of experimental diabetes on the incorporation of amino acids into protein in rat aorta, *Horm. Metab. Res.*, **11**, 384–388.
[30] Grainger, D. J. and Metcalfe, J. C. (1995). A pivotal role for TGF-$\beta$ in atherogenesis? *Biol. Rev. Cambridge Phil. Society*, **70**, 571–596.
[31] Creighton, W. M., Taylor, A. J., Dichek, D. A., Dong, G., Roberts, A. B., Schulick, A. H., Mannam, P. and Virmani, R. (1997). Regional variability in the time course of TGF-beta 1 expression, cellular proliferation and extracellular matrix expansion following arterial injury, *Growth Factors*, **14**, 297–306.
[32] Rasmussen, L. M., Wolf, Y. G. and Rouslanhti, E. (1995). Vascular smooth muscle cells from injured rat aortas display elevated matrix production associated with transforming growth factor-$\beta$ activity, *Am. J. Pathol.*, **147**, 1041–1048.

